# Activation of Sirt1/FXR Signaling Pathway Attenuates Triptolide-Induced Hepatotoxicity in Rats

**DOI:** 10.3389/fphar.2017.00260

**Published:** 2017-05-09

**Authors:** Jing Yang, Lixin Sun, Lu Wang, Hozeifa M. Hassan, Xuan Wang, Phillip B. Hylemon, Tao Wang, Huiping Zhou, Luyong Zhang, Zhenzhou Jiang

**Affiliations:** ^1^Jiangsu Key Laboratory of Drug Screening, China Pharmaceutical UniversityNanjing, China; ^2^Department of Microbiology and Immunology, Virginia Commonwealth University, RichmondVA, USA; ^3^McGuire Veterans Affairs Medical Center, RichmondVA, USA; ^4^Jiangsu Center for Pharmacodynamics Research and Evaluation, China Pharmaceutical UniversityNanjing, China; ^5^State Key Laboratory of Natural Medicines, China Pharmaceutical UniversityNanjing, China; ^6^Key Laboratory of Drug Quality Control and Pharmacovigilance, China Pharmaceutical University, Ministry of EducationNanjing, China; ^7^Jiangsu Key Laboratory of TCM Evaluation and Translational Research, China Pharmaceutical UniversityNanjing, China

**Keywords:** triptolide, hepatotoxicity, bile acid metabolism, hepatic gluconeogenesis, farnesoid X receptor, Sirtuin 1

## Abstract

Triptolide (TP), a diterpenoid isolated from *Tripterygium wilfordii* Hook F, has an excellent pharmacological profile of immunosuppression and anti-tumor activities, but its clinical applications are severely restricted due to its severe and cumulative toxicities. The farnesoid X receptor (FXR) is the master bile acid nuclear receptor and plays an important role in maintaining hepatic metabolism homeostasis. Hepatic Sirtuin (Sirt1) is a key regulator of the FXR signaling pathway and hepatic metabolism homeostasis. The aims of this study were to determine whether Sirt1/FXR signaling pathway plays a critical role in TP-induced hepatotoxicity. Our study revealed that the intragastric administration of TP (400 μg/kg body weight) for 28 consecutive days increased bile acid accumulation, suppressed hepatic gluconeogenesis in rats. The expression of bile acid transporter BSEP was significantly reduced and cholesterol 7α-hydroxylase (CYP7A1) was markedly increased in the TP-treated group, whereas the genes responsible for hepatic gluconeogenesis were suppressed in the TP-treated group. TP also modulated the FXR and Sirt1 by decreasing its expression both *in vitro* and *in vivo*. The Sirt1 agonist SRT1720 and the FXR agonist obeticholic acid (OCA) were used both *in vivo* and *in vitro*. The remarkable liver damage induced by TP was attenuated by treatment with either SRT1720 or OCA, as reflected by decreased levels of serum total bile acids and alkaline phosphatase and increased glucose levels. Meanwhile, SRT1720 significantly alleviated TP-induced FXR suppression and FXR-targets involved in hepatic lipid and glucose metabolism. Based on these results, we conclude that Sirt1/FXR inactivation plays a critical role in TP-induced hepatotoxicity. Moreover, Sirt1/FXR axis represents a novel therapeutic target that could potentially ameliorate TP-induced hepatotoxicity.

## Introduction

The diterpenoid triepoxide triptolide (TP) is the major active component that is extracted from the traditional Chinese medicine, *Tripterygium wilfordii* Hook F (TWHF). TP has multiple pharmacological effects, including anti-inflammatory, immunosuppressive and anti-proliferative activities (Zhou et al., 2012; Ziaei and Halaby, 2016). TP is commonly used to treat certain autoimmune and inflammatory disorders. Minnelide, a water-soluble analog of TP and a synthetic pro-drug, is being studied as a potent chemotherapeutic drug for pancreatic cancer (Rivard et al., 2014; Banerjee and Saluja, 2015). However, due to its narrow therapeutic window and severe toxicities, especially hepatotoxicity, the clinical applications of TP are limited (Zhou et al., 1999). Previous studies have indicated that the TP-induced hepatotoxicity is possibly associated with increased lipid peroxidation (Jiang et al., 2016), DNA damage, mitochondrial impairment (Yao et al., 2008; Fu et al., 2011, 2013) or Th17/Treg imbalance (Wang et al., 2014). However, the exact cellular/molecular mechanisms by which TP induces hepatotoxicity remain unclear. There is an urgent need to develop novel therapeutic strategies to prevent or counteract the TP-induced hepatotoxicity.

The farnesoid X receptor (FXR) is the master nuclear receptor for bile acids and plays an important role in maintaining hepatic bile acid homeostasis through activation of the small heterodimer partner (SHP), which represses the expression of CYP7A1 (Noel et al., 2016). Additionally, FXR also positively regulates essential bile acid transporters such as the ATP-binding cassette, sub-family B member 11 (ABCB11; BSEP) (Chen et al., 2016). Moreover, FXR is involved not only in the regulation of bile acid metabolism, but also in the modulation of glucose metabolism (Stayrook et al., 2005; Watanabe et al., 2006). FXR agonists or bile acids induced a rapid down-regulation of the rate-limiting gluconeogenic genes PEP carboxykinase (PEPCK) and glucose-6-phophatase (G6PC) in the liver (Stayrook et al., 2005; Cao et al., 2010). In addition, studies from several laboratories have shown that FXR is a possible target protein that mediates drug-induced liver injury (DILI) and FXR antagonism may help in the development of DILI (Cariou, 2008). Furthermore, activation of FXR by its agonist GW4064 or by obeticholic acid (OCA) attenuated some forms of DILI (Liu et al., 2003; Mudaliar et al., 2013; Jin et al., 2015). However, activation of AMP-activated protein kinase (AMPK) directly phosphorylates FXR and inhibits its activation and subsequently down-regulates the expression of FXR-targeted genes, which is associated with liver injury under cholestatic conditions (Lien et al., 2014). Consequently, reactivating FXR may be a potential therapeutic strategy for the treatment of DILI.

Sirtuin 1 (Sirt1), a class III NAD+-dependent histone deacetylase, is involved in lipid, glucose and bile acid metabolism. Because it lacks a DNA-binding domain, Sirt1 is recruited to target promoters by sequence-specific transcription factors like FXR. Deacetylation of the transcription factors by Sirt1 results in alterations in gene transcription. Not surprisingly, Sirt1 modulates the FXR-stimulated transcriptional signaling by deacetylation of this nuclear receptor and neighboring histones that strictly control the target gene transcription (Kemper et al., 2009; Garcia-Rodriguez et al., 2014). Accumulating evidence suggests that activation of AMPK leads to increased cellular NAD+ levels, which subsequently modulates the Sirt1 signaling (Canto et al., 2009).

In this study, we tested the hypothesis that TP suppresses the FXR pathway by reducing the Sirt1 activity, which leads to the dysregulation of the key genes involved in hepatic metabolism including SHP, BSEP, PEPCK, G6PC, and CYP7A1 and eventually induces liver injury. These results identified a potential mechanism associated with TP-induced liver injury and potential targets that could be applied to prevent or ameliorate TP-induced liver injury.

## Materials and Methods

### Materials

Triptolide (>98%, HPLC) was a gift from the Institute of Dermatology, Chinese Academy of Medical Sciences and Peking Union Medical College (Nanjing, China). GW4064 was purchased from Sigma Chemical Co. (St. Louis, MO, USA). OCA was obtained from Shanghai Houpu Chemical Co. (Shanghai, China). SRT1720 was purchased from Shanghai Yongcan Chemical Co. (Shanghai, China). RIPA lysis buffer was purchased from Beyotime (Nanjing, China). The Bio-Rad protein assay reagent, Precision Plus Protein Kaleidoscope Standards, 4 × sample buffer and SYBR Green Supermix were from Bio-Rad (Hercules, CA, USA). The primary antibodies against FXR (sc-13063), SHP (sc-30169), CYP7A1 (sc-25536), PEPCK (sc-32879), and β-actin (sc-47778) were from Santa Cruz Biotechnology (Santa Cruz, CA, USA). The phospho-AMPK (2535s), AMPK (2532), and Sirt1 (9475s) primary antibodies were from Cell Signaling Technology (Danvers, MA, USA).

### Isolation and Culture of Primary Rat Hepatocytes

Primary rat hepatocytes were prepared from female Wister rats and seeded in collagen-coated 60 mm dish at 2 × 10^6^ cells per dish as previously described (Studer et al., 2012). The cells were cultured in serum-free Williams’ E medium containing dexamethasone (0.1 μM), penicillin (100 units/ml), and thyroxine (1 μM).

### Animal Studies

Wistar rats (female, 180–200 g) were purchased from the SLRC Laboratory Animal Company (Shanghai, China). All experiments and procedures involving rats were approved by the Ethical Committee of China Pharmaceutical University, and the Laboratory Animal Management Committee of Jiangsu Province (Approval No.: 2110748) and were conducted in accordance with all applicable regulations.

The rats were raised in an aseptic facility (temperature: 24 ± 2°C, relative humidity: 40 ± 10%) with 12-h light/dark cycle and given free access to food and water. The animals were acclimated for one week before the experiments. The rats were randomly divided into four groups (*n* = 6/group): (1) control; (2) TP (400 μg/kg/day); (3) OCA (15 mg/kg/day) +TP (400 μg/kg/day); and (4) SRT1720 (10 mg/kg/day) +TP (400 μg/kg/day). The rats were fed a standard rat chow and gavaged daily with control solution (0.5% CMC-Na), or TP for 4 weeks. Then, all rats were fasted for 12 h, and blood samples and liver tissues were collected for analysis.

### RNA Extraction and Real-Time PCR

The total RNA was isolated from the liver tissues or primary hepatocytes using TRIzol Reagent (Carlsbad, CA, USA) and reversed transcribed into cDNA using the High-capacity cDNA Reverse Transcription Kit (Waltham, MA, USA). The mRNA levels of FXR, SHP, CYP7A1, BSEP, PEPCK, G6PC, and Sirt1 were determined using real-time PCR with Thermo Hygreen Supermix reagents and normalized to GAPDH as the internal control. The primer sequences used were listed in Supplementary Table S1.

### Western Blot Analysis

The total cellular proteins were collected by lysis with RIPA lysis buffer. The protein concentration was determined using the Bio-Rad protein assay reagent. The total proteins (100 μg) were resolved on 10% SDS-PAGE gel and transferred to nitrocellulose membranes. The immunoreactive bands were detected using horseradish peroxidase-conjugated secondary antibodies and ECL reagents. The bands were analyzed using Quantity One.

### Statistical Analysis

The data are expressed as the means ± SEM. One-way analysis of variance (ANOVA) and Dunnett’s *t*-test was used to evaluate the differences among the various groups using GraphPad Prism 5.0 (San Diego, CA, USA). The differences were considered statistically significant at *p* < 0.05.

## Results

### TP Inhibited FXR Pathway in Primary Rat Hepatocytes

**FIGURE 1:**
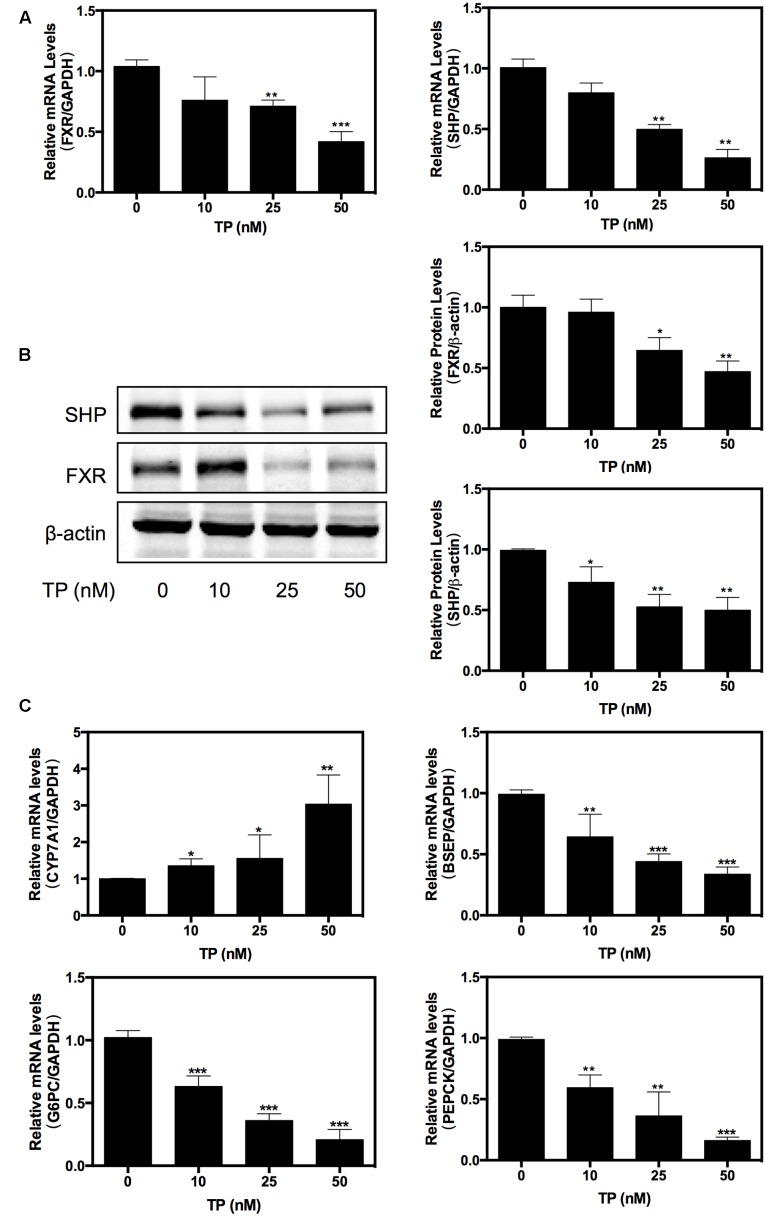
**Triptolide (TP) inhibits farnesoid X receptor (FXR) pathway in primary rat hepatocytes**. Primary rat hepatocytes were treated with TP at various concentrations (0–50 nM) for 8 h. **(A)** The mRNA level of FXR was measured using real-time RT-PCR and normalized to GAPDH as an internal control. **(B)** The protein expression level of FXR was determined using western blot analysis and normalized to β-actin as a loading control. **(C)** The mRNA levels of FXR-target genes were measured using real-time RT-PCR and normalized to GAPDH as an internal control. Statistical significance relative to vehicle control: ^∗^*p* < 0.05, ^∗∗^*p* < 0.01, ^∗∗∗^*p* < 0.001.

Farnesoid X receptor is an important regulator of hepatic metabolism. We investigated whether TP had any effect on hepatic FXR expression. Primary rat hepatocytes were treated with various concentrations of TP (0, 10, 25, 50 nM) for 8 h. As shown in **Figures 1A–B**, at 25 or 50 nM concentration, TP dramatically inhibited expression of FXR and SHP at both the mRNA and protein levels in primary rat hepatocytes. Since FXR plays a critical role in regulating bile acid and glucose metabolism, we further examined the expression of the key genes involved in bile acid synthesis and transport as well as hepatic gluconeogenesis. The results shown in **Figure 1C** indicated that TP dose-dependently up-regulated the mRNA expression of CYP7A1, but it suppressed the mRNA expression of BSEP, PEPCK, and G6PC. These results suggest that inhibition of FXR expression may contribute to TP-induced dysregulation of hepatic metabolism and hepatotoxicity.

### TP Induced FXR Suppression via Sirt1 *In Vitro*

**FIGURE 2:**
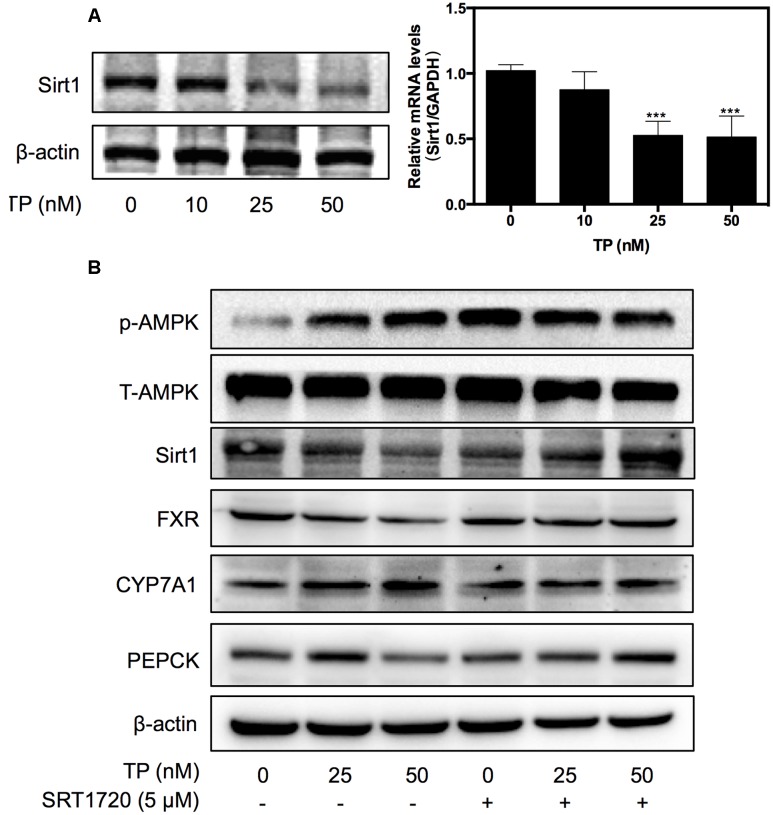
**Effect of Sirtuin (Sirt1) in TP-induced FXR suppression**. **(A)** Primary rat hepatocytes were treated with TP for 8 h. Sirt1 expression was determined using real-time RT-PCR and western blot analysis. **(B)** Primary rat hepatocytes were pretreated with a Sirt1 agonist, SRT1720 (5 μM), for one hour and then treated with TP for 8 h. The specific protein levels were determined using western blot analysis and normalized to β-actin as a loading control. Statistical significance relative to vehicle control, ^∗^*p* < 0.05, ^∗∗^*p* < 0.01, ^∗∗∗^*p* < 0.001; and statistical significance relative to the corresponding treatment group without SRT1720, ^#^*p* < 0.05, ^##^*p* < 0.01, ^###^*p* < 0.001.

Sirt1 is a NAD+-dependent deacetylase, which deacetylates histone and non-histone proteins such as FXR. To identify the upstream signaling pathway involved in TP-induced FXR inactivation, the effect of TP on Sirt1 expression was examined. As shown in **Figure 2A**, TP suppressed Sirt1 expression at both the mRNA and protein levels.

Based on the observations that TP suppressed both FXR and Sirt1 expression, we used a synthetic Sirt1 agonist, SRT1720, to investigate whether activation of Sirt1 could have a protective effect against TP-induced liver injury. As shown in **Figure 2B**, SRT1720 (5 μM) significantly alleviated TP-induced down-regulation of FXR protein expression, but had no effect on TP-induced AMPK activation. To further assess whether activation of Sirt1 also prevents TP-induced dysregulation of the expression of key genes involved in hepatic lipid and glucose metabolism, primary rat hepatocytes were pretreated with SRT1720 and then treated with TP. The protein levels of the FXR-targets, CYP7A1 and PEPCK, were measured using western blotting. As shown in **Figure 2B**, CYP7A1, a rate-limiting enzyme of bile acid synthesis, was markedly increased by TP and this effect was reduced by pretreatment with SRT1720. TP-induced inhibition of PEPCK protein expression, a key regulator of hepatic gluconeogenesis, was also blocked by SRT1720. These results indicated that Sirt1 inhibition was responsible for TP-induced FXR suppression and disruption of hepatic metabolism.

### Activation of FXR Ameliorates TP-Induced Hepatotoxicity

**FIGURE 3:**
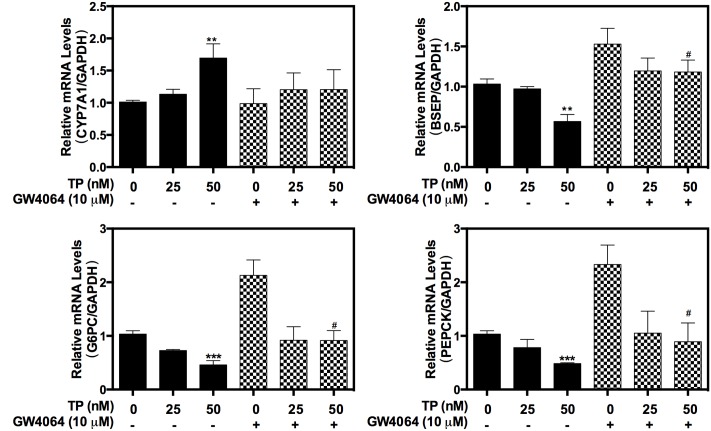
**Effect of GW4064 on TP-induced hepatotoxicity in primary rat hepatocytes**. Primary rat hepatocytes were pretreated with the FXR agonist, GW4064 (10 μM), for one hour and then treated with TP for 8 h. The mRNA levels of FXR-target genes were determined using real-time RT-PCR and normalized to GAPDH as an internal control. Statistical significance relative to vehicle control, ^∗^*p* < 0.05, ^∗∗^*p* < 0.01, ^∗∗∗^*p* < 0.001; and statistical significance relative to the corresponding treated groups without GW4046, ^#^*p* < 0.05, ^##^*p* < 0.01, ^###^*p* < 0.001.

In order to further delineate the role of FXR in TP-induced hepatotoxicity, a synthetic FXR agonist, GW4064, was used. TP-induced dysregulation of the expression of hepatic bile acid transporter BSEP and the key genes of hepatic gluconeogenesis, PEPCK and G6PC as well as CYP7A1, were significantly inhibited by GW4064 (**Figure 3**). These results indicated that activation of FXR could reduce TP-induced hepatotoxicity.

**FIGURE 4:**
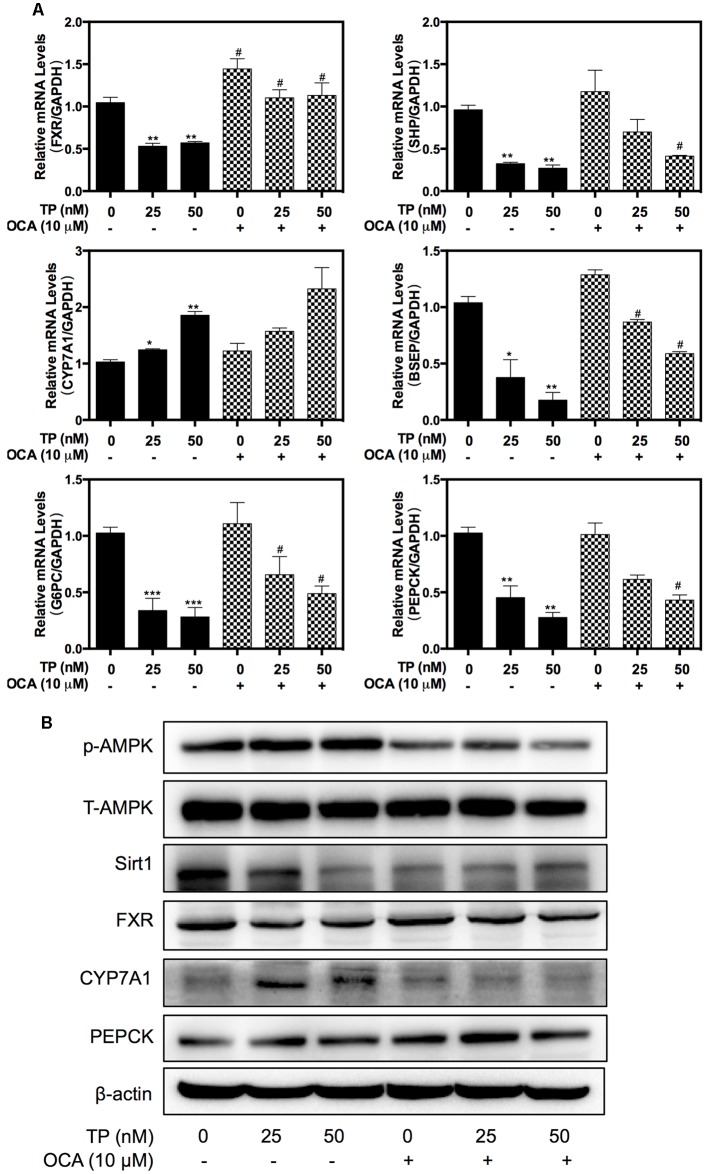
**Role of FXR in TP-induced hepatotoxicity**. Primary rat hepatocytes were pretreated with the FXR agonist, obeticholic acid (OCA) (10 μM), for one hour and then treated with TP for 8 h. **(A)** The mRNA levels of FXR-target genes were determined using real-time RT-PCR and normalized to GAPDH as an internal control. **(B)** The target protein levels were determined using western blot analysis and normalized to β-actin as a loading control. Statistical significance relative to vehicle control, ^∗^*p* < 0.05, ^∗∗^*p* < 0.01, ^∗∗∗^*p* < 0.001; and statistical significance relative to the corresponding treated groups without OCA, ^#^*p* < 0.05, ^##^*p* < 0.01, ^###^*p* < 0.001.

To further evaluate the protective role of FXR against TP-induced hepatotoxicity, primary rat hepatocytes were pretreated with OCA, which is a semi-synthetic bile acid analog and the first FXR agonist in a human clinical trial, and then treated with TP. As shown in **Figure 4A**, OCA partially attenuated the TP-induced dys-regulation of the mRNA expression of BSEP, CYP7A1 and SHP. Western blots further indicated that OCA prevented TP-induced down-regulation of FXR protein expression (**Figure 4B**). However, OCA had no effect on TP-induced down-regulation Sirt 1 expression. For the data showed in **Figures 3**, **4**, OCA may have stronger effects on FXR signaling pathway. The above data indicate that the loss of hepatic FXR was responsible for TP-induced disorders of the bile acid and glucose metabolism and the subsequent hepatotoxicity.

### Protective Effect of Sirt1 on TP-Induced Hepatotoxicity in Rats

**FIGURE 5:**
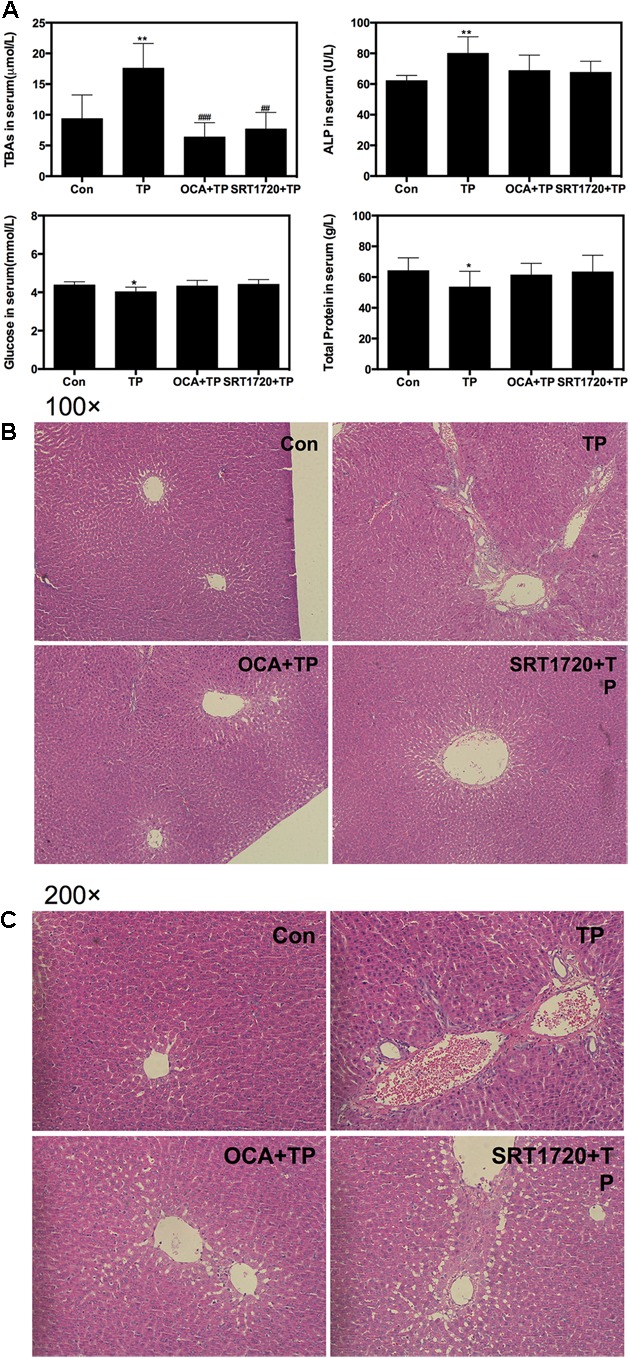
**Protective effect of Sirt1 on TP-induced hepatotoxicity in rat model**. Female Wistar rats were treated as previously described in the Materials and Methods. **(A)** The serum levels of TBA, ALP, glucose and total protein levels were measured. **(B,C)** Representative images of the HE staining of the rat liver sections. The images were recorded using an Olympus microscope equipped with a 100× lens and 200× lens using an image recorder.

To investigate whether FXR activation would protect against TP-induced hepatotoxicity *in vivo*, we established an animal model of liver injury by oral administration of TP for 4 weeks and evaluated the protective effects of SRT1720 and OCA on TP-induced hepatotoxicity. Body weight was decreased, but not significantly different in the TP-treated group compared to the control group. However, as shown in **Figure 5A**, TP markedly increased the total bile acids (TBAs) and ALP levels in the serum, indicating cholestatic liver injury. Co-administration of OCA or SRT1720 reduced TP-induced hepatic injury. In addition, the serum glucose and total protein levels were decreased in the TP-treated group compared to the control group, but these effects were prevented in the OCA+TP and SRT1720+TP groups. The hepatic histopathological analysis revealed extensive hepatic parenchymal necrosis, vacuolated congestion and bile duct proliferation in the group treated with TP alone and these effects were significantly decreased when TP was combined with either OCA or SRT1720 (**Figures 5B,C** and **Supplementary Figure S1**).

**FIGURE 6:**
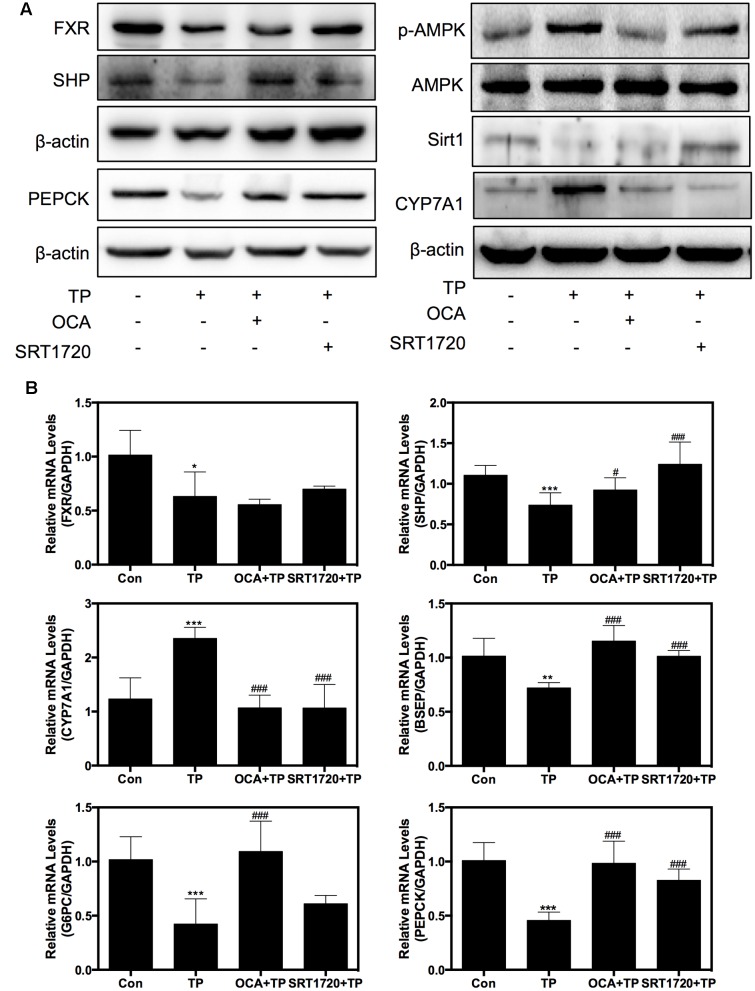
**Regulation of the expression of proteins and genes involved in TP-induced hepatotoxicity by SRT1720**. Female Wistar rats were treated as previously described in the Section “Materials and Methods”. **(A)** Representative immunoblots against p-AMPK, T-AMPK, Sirt1, FXR, and FXR-targets in the liver lysates are shown and normalized to β-actin as a loading control. **(B)** The relative mRNA levels of the relevant genes were determined using real-time RT-PCR and normalized to GAPDH as an internal control. Statistical significance relative to vehicle control, ^∗^*p* < 0.05, ^∗∗^*p* < 0.01, ^∗∗∗^*p* < 0.001; and statistical significance relative to the TP treated group, ^#^*p* < 0.05, ^##^*p* < 0.01, ^###^*p* < 0.001.

Western blot analysis further indicated that TP co-treatment with either OCA or SRT1720 remarkably inhibited the TP-induced FXR suppression in the rat model (**Figure 6A**). OCA suppressed the AMPK phosphorylation and activated the FXR pathway, but SRT1720 did not affect the AMPK activation. The protein expression of specific FXR-targets including SHP and PEPCK was increased by the OCA or SRT1720 co-treatment. The CYP7A1 expression that had been increased by TP was suppressed by co-treatment with OCA or SRT1720. Similar to the results in the primary rat hepatocytes, OCA and SRT1720 also inhibited the TP-induced FXR suppression and the expression of key genes involved in bile acid synthesis and transport as well as hepatic gluconeogenesis at their mRNA levels (**Figure 6B**).

Taken together, these findings indicated that co-treatment with OCA or SRT1720 provided protection against TP-induced hepatotoxicity and supported our hypothesis that FXR activation plays a critical role in relieving TP-induced hepatotoxicity.

## Discussion

**FIGURE 7:**
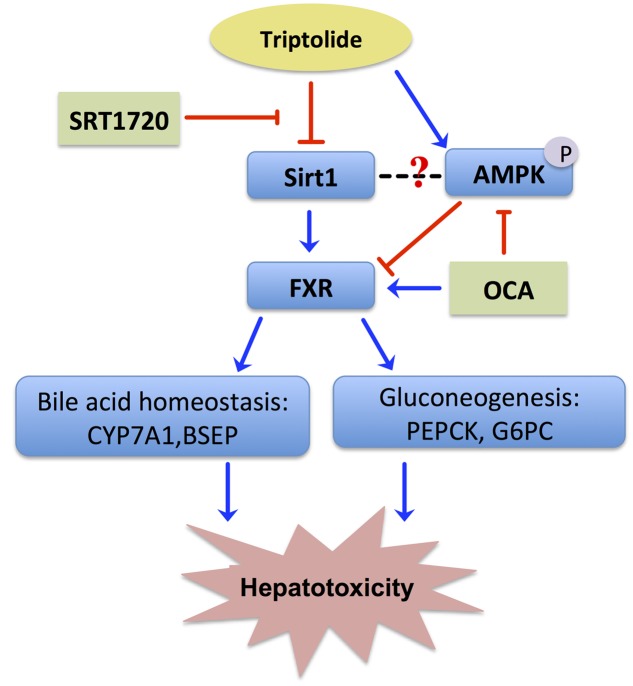
**Proposed signaling pathways involved in TP-induced hepatotoxicity**. TP suppressed the FXR/SHP pathway *via* inhibiting the Sirt1 activity, which led to dysregulation of the expression of FXR-targeted genes involved in bile acid homeostasis and gluconeogenesis. Both Sirt1 agonist, SRT1720, and FXR agonist, OCA, reduced TP-induced disruption of hepatic metabolism and liver injury.

Drug-induced liver injury is the most frequent key cause for termination of drug development during or after preclinical studies and for drug withdrawal from the market (Schadt et al., 2016). Despite exhibiting excellent immunosuppressive and anti-tumor activities, TP has been greatly restricted in clinical application on the basis of a severe and cumulative hepatotoxicity. The toxicity of TP is closely related to its dose and duration. It has been reported that TP-induced hepatotoxicity occurred frequently in cases of either a single high dose or long-term low doses. Based on previous studies, a 400 μg /kg dose of TP administered orally for 28 days was used as the model for the evaluation of TP-induced toxicity in this study (Li et al., 2014). In addition, our recent studies indicated that TP induced more severe injury in female rats (Liu et al., 2015; Jiang et al., 2016). Similar to the results reported in the literature, TP increased serum total bile acid and ALP levels as compared to the control group in Wistar rats, indicating that TP by oral administration for 28 days significantly induced hepatotoxicity. However, the cellular mechanisms underlying TP-induced hepatotoxicity remain unclear. Our previous genome-wide microarray study indicated that TP alters the expression of 3329 genes that are involved in glucose metabolism and cellular stress pathways (Wang et al., 2013). In this study, we found that TP inhibited the Sirt1/FXR signaling pathway and caused dysregulation of bile acid homeostasis and hepatic gluconeogenesis (**Figure 7**). Our results indicate that inactivation of FXR may contribute to TP-induced hepatotoxicity. Furthermore, activation of Sirt1 through up-regulating FXR signaling pathway could have a protective effect against TP-induced disruption of hepatic metabolism.

Sirt1 is a critical regulatory factor in the hepatic glucose metabolic processes. Hepatic deletion of Sirt1 leads to mild hypoglycemia, increased glucose tolerance, decreased hepatic glucose production, decreased serum cholesterol and increased hepatic content of free fatty acids and cholesterol, all of which can be reversed by Sirt1 overexpression (Rodgers and Puigserver, 2007). The current study showed that the rats treated with TP had mild hypoglycemia, which was correlated with changes of the protein expression levels involved in hepatic gluconeogenesis (**Figure 6**). Importantly, many of these changes were reversed by SRT1720. On the other hand, hepatic deletion of Sirt1 leads to dysfunctions in bile acid metabolism through down-regulation of FXR signaling and the development of cholesterol gallstones on a lithogenic diet (Purushotham et al., 2012). Similarly, TP caused the accumulation of hepatic bile acids and increased serum ALP, which was blocked by SRT1720 (**Figure 5**).

FXR plays a crucial role in hepatic bile acid and glucose metabolism, hepatic regeneration, and the stress response to hepatotoxins and is frequently decreased in liver damage (Cariou, 2008; Lee F.Y. et al., 2010; Vaquero et al., 2013). FXR activation by agonists may represent an attractive therapeutic concept in cholestasis. GW4064 is a selective, non-steroidal FXR agonist (EC50 = 65 nM), while OCA is a potent and selective FXR agonist (EC50 = 99 nM). Now, OCA is used as a drug to treat primary biliary cholangitis, and is undergoing development for several other liver diseases and related disorders. This current study further demonstrated that dysfunction of FXR plays a key role in TP-induced hepatotoxicity. TP inhibited FXR expression and activity by down-regulating or inactivating hepatic Sirt1, resulting in disruption of bile acid and glucose metabolism and hepatic damage. It has been well-characterized that hepatic Sirt1 can modulate the FXR signaling pathway *via* different mechanisms. First, Sirt1 directly regulates the deacetylation of FXR, promotes its stability, inhibits its DNA-binding ability and transactivation activity (Kemper et al., 2009). Second, hepatic SIRT1 indirectly modulates FXR activity through HNF1α. In a liver-specific Sirt1 knockout mouse model, Sirt1 modulates the FXR signaling by down-regulating the HNF1α recruitment to the FXR promoter and the expression of FXR (Purushotham et al., 2012). Third, hepatic Sirt1 may also indirectly increase the mRNA levels of FXR by activating PGC-1α (Zhang et al., 2004). Hepatic deletion of Sirt1 decreased co-activation of PGC-1α (Purushotham et al., 2009). Finally, hepatic Sirt1 may indirectly regulate the transactivation activity of FXR by stimulating the biosynthesis of bile acids. The rate-limiting enzyme for bile acid synthesis, CYP7A1, is involved in cholesterol efflux and degradation, which is down-regulated by Sirt1 shRNA and induced by Sirt1 overexpression in the fasted state (Rodgers and Puigserver, 2007). In contrast, several lines of evidence suggest that the FXR-SHP pathway lies upstream of Sirt1 and regulates this protein via the p53/miR-34a pathway (Lee J. et al., 2010). Therefore, Sirt1 and the FXR signaling pathway mutually interact at multiple levels in response to various stimuli to coordinately regulate hepatic bile acid, cholesterol and glucose homeostasis. In this study, we found that TP-induced down-regulation of FXR protein expression was also blocked by SRT1720 (**Figure 2**). We will further explore the exact mechanism by which TP modulates the Sirt1 and FXR signaling pathway.

AMP-activated protein kinase is another important player in regulating hepatic metabolism. In this study, we found that SRT1720 had no effect on p-AMPK/AMPK. These results suggest either that AMPK is located upstream of Sirt1 or that the AMPK-mediated regulation of FXR is independent of Sirt1 (**Figure 2**). Previous studies reported that activation of AMPK increases the NAD+ levels, which leads to activation of Sirt1 and deacetylation of its down-stream targets such as PGC-1α (Chau et al., 2010; Kim et al., 2013), FOXO1 (Canto et al., 2009), FXR (Garcia-Rodriguez et al., 2014), and PPARα (Kang et al., 2016). However, the current study indicated that TP simultaneously activated the phosphorylation of AMPK and suppressed the Sirt1 expression. Several studies have clearly demonstrated that AMPK negatively regulates Sirt1 expression or has no effect on Sirt1expression (Zu et al., 2010; Lee et al., 2012; Gollavilli et al., 2015). Lien reported that metformin induces activation of AMPK, which directly phosphorylates and regulates the FXR transcriptional activity and subsequently contributes to hepatocyte injury (Lien et al., 2014). Our recent study also showed that 17α-ethinylestradiol (EE)-induced activation and nuclear translocation of AMPKα1 plays a critical role in EE-induced cholestasis via down-regulation of the expression of FXR and bile acid receptors (Li et al., 2016). In the current study, we also found that rapidly and dose-dependently induced AMPK activation (**Figure 4B**). A semi-synthetic bile acid analog, OCA, not only activates FXR and improves bile acid and glucose metabolism, but also inhibits AMPK activation. OCA significantly inhibited TP-induced hepatotoxicity (**Figures 4**, **6**). These findings further confirmed that AMPK also plays an important role in the TP-induced down-regulation of FXR. We will further evaluate whether activation of AMPK had any effect on the Sirt1expression in TP-induced hepatotoxicity in the rat model.

## Conclusion

Sirt1 is an endogenous activator of FXR in hepatocytes and often is decreased in liver injury. The present study demonstrated that TP-induced down-regulation of hepatic Sirt1 contributed to the reduced hepatic FXR levels and subsequently led to disruption of hepatic bile acid and glucose metabolism. Moreover, this Sirt1-FXR axis represents an important signaling pathway that may have the potential to ameliorate DILI in humans.

## Author Contributions

JY, HZ, LZ, and ZJ designed the experiments. JY, LS, LW, and XW performed the experiments. JY, TW, PH, HZ, and ZJ analyzed the data. JY, HH, HZ, LZ, and ZJ wrote the paper. All authors contributed to the editing of the paper and to scientific discussions.

## Conflict of Interest Statement

The authors declare that the research was conducted in the absence of any commercial or financial relationships that could be construed as a potential conflict of interest.

## Supplementary Material

FIGURE S1 **Triptolide (TP)-induced hepatic injury in rat model**. Female Wistar rats were treated as previously described in the Materials and Methods. The images of the rat liver were recorded.
