# Supplementary figures and images for: Activation of Sirt1/FXR Signaling Pathway Attenuates Triptolide-Induced Hepatotoxicity in Rats
Source: Front Pharmacol. 2017 May 9;8:260. doi: 10.3389/fphar.2017.00260 (PMC5422577; doi:10.3389/fphar.2017.00260)

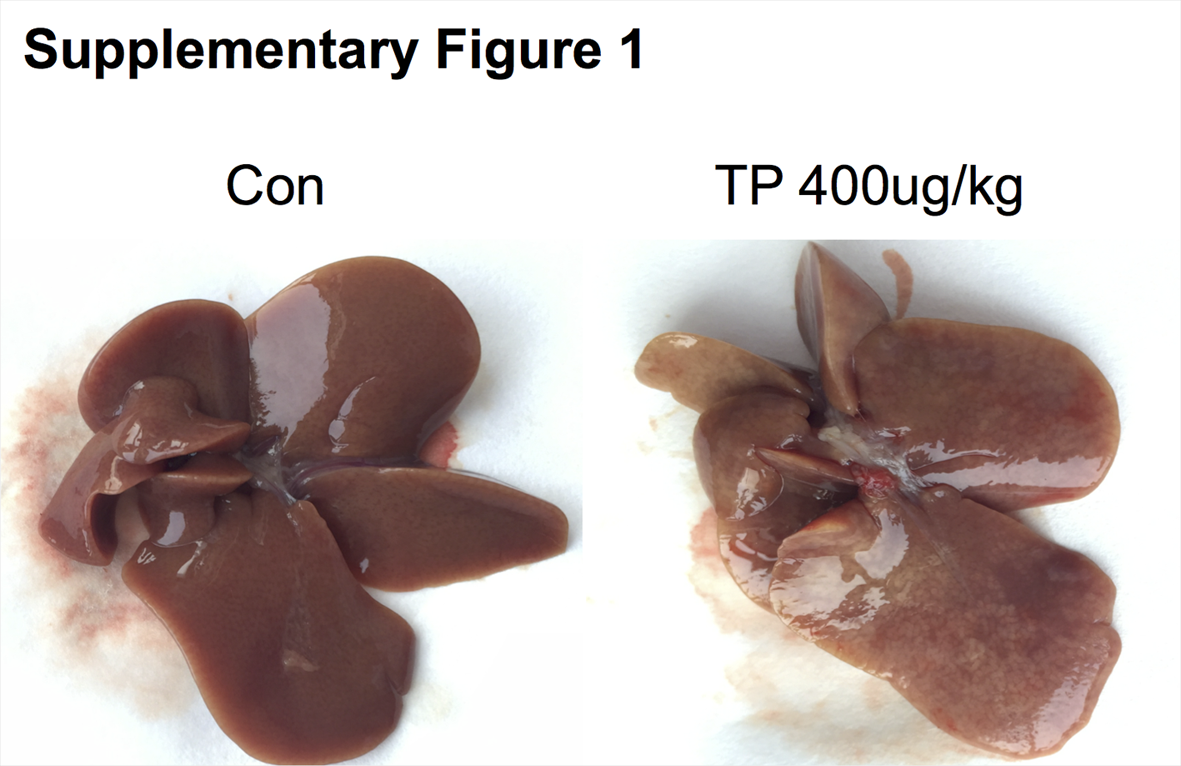

Supplement: FIGURE S1 — Triptolide (TP)-induced hepatic injury in rat model. Female Wistar rats were treated as previously described in the Materials and Methods. The images of the rat liver were recorded. [file Image_1.TIFF]
