# Supplementary material for: Activation of Sirt1/FXR Signaling Pathway Attenuates Triptolide-Induced Hepatotoxicity in Rats
Source: Front Pharmacol. 2017 May 9;8:260. doi: 10.3389/fphar.2017.00260 (PMC5422577; doi:10.3389/fphar.2017.00260)
Supplement: Supplementary file 2 [file Table_1.DOCX]

Supplementary Table 1. The primer sequences used for real-time PCR assay in rats.

| Gene name | Primers (5’-3’) |
| --- | --- |
| GAPDH | F: ATGGAGAAGGCTGGGGCTCACCT |
|  | R: AGCCCTTCCACGATGCCAAAGTTGT |
| FXR | F: TGGACTCATACGCAAACAGAGA |
|  | R: GTCTGAAACCCTGGAAGTCTTTT |
| SHP | F: ACCTGCAACAGGAGGCTCACT |
|  | R: TGGAAGCCATGAGGAGGATTC |
| CYP7A1 | F: CAGGGAGATGCTCTGTGTTCA |
|  | R: AGGCATACATCCCTTCCGTGA |
| BSEP | F: CAACGCATTGCTATTGCTCG |
|  | R: CTTCTGGATGGTGGACAAACG |
| PEPCK | F: TGTTGGCTGGCTCTCACTG |
|  | R: ACTTTTGGGGATGGGCAC |
| G6PC | F: CTACCTTGCGGCTCACTTTC |
|  | R: ATCCAAGTGCGAAACCAAAC |
| Sirt1 | F: ACAACCTCCTGTTGGCTGATG |
|  | R: GCTTGCGTGTGATGCTCTGT |
